# Visual and Quantitative Analysis Methods of Respiratory Patterns for Respiratory Gated PET/CT

**DOI:** 10.1155/2016/7862539

**Published:** 2016-10-31

**Authors:** Hye Joo Son, Young Jin Jeong, Hyun Jin Yoon, Jong-Hwan Park, Do-Young Kang

**Affiliations:** ^1^Department of Nuclear Medicine, Dong-A University Medical Center, Dong-A University College of Medicine, Busan, Republic of Korea; ^2^The Dong-A Anti-Aging Research Institute, Dong-A University, Busan, Republic of Korea

## Abstract

We integrated visual and quantitative methods for analyzing the stability of respiration using four methods: phase space diagrams, Fourier spectra, Poincaré maps, and Lyapunov exponents. Respiratory patterns of 139 patients were grouped based on the combination of the regularity of amplitude, period, and baseline positions. Visual grading was done by inspecting the shape of diagram and classified into two states: regular and irregular. Quantitation was done by measuring standard deviation of* x* and* v* coordinates of Poincaré map (SD_*x*_, SD_*v*_) or the height of the fundamental peak (*A*_1_) in Fourier spectrum or calculating the difference between maximal upward and downward drift. Each group showed characteristic pattern on visual analysis. There was difference of quantitative parameters (SD_*x*_, SD_*v*_,* A*_1_, and MUD-MDD) among four groups (one way ANOVA, *p* = 0.0001 for MUD-MDD, SD_*x*_, and SD_*v*_, *p* = 0.0002 for* A*_1_). In ROC analysis, the cutoff values were 0.11 for SD_*x*_ (AUC: 0.982, *p* < 0.0001), 0.062 for SD_*v*_ (AUC: 0.847, *p* < 0.0001), 0.117 for* A*_1_ (AUC: 0.876, *p* < 0.0001), and 0.349 for MUD-MDD (AUC: 0.948, *p* < 0.0001). This is the first study to analyze multiple aspects of respiration using various mathematical constructs and provides quantitative indices of respiratory stability and determining quantitative cutoff value for differentiating regular and irregular respiration.

## 1. Introduction

One of the key challenges associated with imaging of thoracic tumors using current PET/CT systems is respiratory motion [1]. Respiration results in blurring of a tumor over multiple respiration cycles and in underestimation of metabolic uptake as well as in overestimation of the tumor volume [2]. Respiratory gated PET/CT correlates the PET data acquisition with the breathing phase and enables multiple PET images associated with different respiration phases to be reconstructed as distinct scans [3]. It reduces the respiratory smearing and enables more accurately defining the tumor volume and improving its standardized uptake value (SUV) [4].

Despite the improved accuracy of respiratory gated PET/CT, the gains are patient-specific because respiratory patterns are patient-specific. Breathing is a dynamic phenomenon, controlled by complex neurophysiologic feedback and feed-forward coupling mechanisms [5]. The key to successful respiratory gating is a highly stable respiration that enables accurate data binning [3]. Therefore, if patient-specific breathing patterns can be analyzed and evaluated prior to the PET/CT acquisition, personalized motion correction methods can be developed.

Modeling respiratory motion remains a critical issue in the field of radiation therapy. Several authors have investigated the characteristics of the respiratory patterns and their variation during treatment for minimizing the influence of respiratory motion and improving the delivery accuracy. Basic characteristics of respiratory motion were summarized previously [6]. A finite state model has been proposed for representing the respiratory motion using line segments and capturing the cycles in terms of duration, travelled distance, and velocity [7]. Amplitude, period, baseline position, and end-of-inhale and end-of-exhale position of respiration have been analyzed, and a purely periodic model has been suggested; however, the suggested model cannot satisfactorily account for highly irregular respiration [8]. Asymmetric tumor motion has been analyzed in terms of the amplitude of the tumor motion in three directions, the difference in breathing levels during treatment, hysteresis (the difference between the inhalation and exhalation trajectories of the tumor when there is a phase difference), and the amplitude of the tumor motion induced by cardiac motion [9]. Time-amplitude curves of respiration have been decomposed into various subcomponents, such as peak-to-peak amplitude, period, the mean location of end-of-exhale position, and the maximal upward and downward drifts [10]. Several motion scenarios during respiratory gated radiotherapy have been presented, reflecting the differences between simulated and treatment-related CT, such as cycle change, baseline shift, displacement change, and breathing type change (abdominal or chest breathing) [11].

Methodologies presented in the above studies were limited to decomposing the time-amplitude curves into several subcomponents. Herein, we transformed the time-amplitude curves into mathematical constructs such as phase space diagrams, Fourier spectra, Poincaré plots, and Lyapunov exponents. By offering various tools for analysis of respiratory patterns, with each tool having its distinctive merits and limitations, we suggest selecting most suitable methods satisfying the needs of specific clinical situation. Importantly, our studies allow grouping the respiratory patterns into multiple functionally distinct categories and suggest visual and quantitative criteria for classifying regular and irregular respiratory patterns. Finally, our approach integrates visual and quantitative methods for evaluating the respiration stability.

In this study, we introduce visual and quantitative methods for analysis of respiratory patterns during respiratory gated PET/CT. We analyzed respiratory motions induced by free breathing of 139 patients by using phase space diagrams, spectral analysis, Poincaré maps, and Lyapunov exponents. Then, we grouped the patients' breathing patterns with similar motion characteristics into multiple functionally distinct categories. Finally, we compared the different methods in terms of their benefits and limitations.

## 2. Materials and Methods

### 2.1. The Studied Population

From July 2013 to April 2014, 139 patients underwent respiratory gated ^18^F-FDG PET/CT scans for cancer evaluation. Each patient was asked to breathe freely during ^18^F-FDG PET/CT acquisition, without any breathing coaching. The patient-specific information is summarized in Table 1. After fasting for at least 8 hours, the patients were given intravenous injections of 5.2 MBq/kg ^18^F-FDG. PET/CT acquisition started 60 minutes after the radiotracer injection. The use of the data for research purposes was approved and the need for written informed consent was waived by the institutional review board (IRB-15-026).

### 2.2. PET/CT Scanner

The respiratory gated PET/CT protocol consisted of nongated CT and nongated PET along with gated PET (Varian RPM) on predefined beds, followed by gated CT. The PET data were acquired using a Discovery 710 PET/CT scanner (General Electric Medical System, Waukesha, WI, USA). For each patient, a nongated CT scan for attenuation correction was performed with a slice thickness of 3.75 mm, a pitch of 0.969 : 1, a noise index of 25.00, a rotation time of 0.5 s, and at 120 kVp and 80–100 mA, depending on the body weight. After the nongated PET scan, a respiratory gated PET scan over 2-bed position (10 min per each bed) was obtained (phase binning). The respiratory gated PET data were binned into five bins (duration: 120 ms) synchronized with the patient breathing cycle. For respiratory gated images, attenuation correction was performed by using the phase-matched gated CT. Respiratory gated CT scans with cine mode using ultra low dose protocol were performed with a slice thickness of 5 mm, a noise index of 120.00, a rotation time of 0.5 s, a cine time between images of 0.35 s, and at 100 kVp and 10–40 mA, depending on the body weight. The PET images were reconstructed using full 3D iterative reconstruction with point spread function (PSF): a 192 × 192 matrix, 3 iterations, and 16 subsets.

### 2.3. Respiratory Gating System

Respiratory signals were recorded with real time position management (RPM) respiratory gating system (Varian Medical Systems, Palo Alto, CA, USA, software version number 1.7.5). For each patient, the recording duration was 20 min. In the RPM system, a lightweight plastic block with a pair of infrared reflective markers was attached to the patient's abdomen approximately halfway between the xiphoid process and the umbilicus, and its motion was monitored and tracked using an infrared video camera on the PET table. More details about the RPM system can be found in Chang et al. [12].

### 2.4. Analysis Methods of Respiratory Patterns

**Figure 1:**
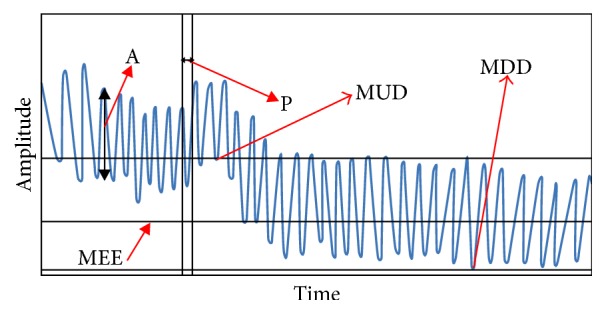
Subcomponents of time-amplitude curve. A: amplitude, P: period, MEE: mean location of end-of-exhale, MUD: maximal upward drift position, and MDD: maximal downward drift position.

Time-amplitude curves of respiratory patterns of 139 patients were observed and divided into four groups by visual analysis based on the combination of the regularity of amplitude and period. Group A showed respiratory pattern that both amplitude and period were regular. Group B showed pattern that amplitude was regular, but period was irregular. Group C showed pattern that amplitude was irregular, but period was regular. Group D showed pattern that both amplitude and period were irregular. Each group is further subdivided depending on the existence of baseline drift. Visual grading of amplitude and period was done by inspecting the shape of phase space diagram or Fourier spectrum and classified into two states: regular and irregular. By inspecting the time-amplitude curve, visual grading of the baseline stability is classified into two states, whether or not baseline drift is present. Then, quantitative evaluation of respiratory stability is done in each group. Regularity of amplitude or period was expressed by measuring standard deviation of *x* (AP direction) and *v* coordinates of Poincaré map (SD_*x*_, SD_*v*_) or measuring the height of the fundamental peak (*A*_1_) in Fourier spectrum. Stability of the baseline position was expressed quantitatively by calculating the difference between maximal upward drift (MUD) position and maximal downward drift (MDD) position (Figure 1) [10]. Less than 5% of respiration cycle was not correlated with the majority of the pattern; this cycle was discarded from analysis.

For each time-amplitude curve, the respiratory signal information was mathematically transformed into phase space diagram, Fourier spectrum, Poincaré map, and Lyapunov exponent.

**Figure 2:**
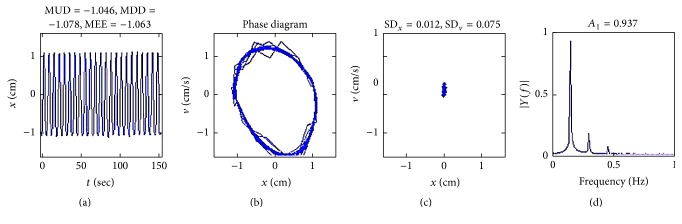
Example of typical regular respiratory pattern: respiratory pattern of phantom. (a) Time-amplitude curve. (b) Phase space diagram. (c) Poincaré map. (d) Frequency spectrum.

A phase space diagram depicts the velocity, *v*(*t*), as a function of the displacement, *x*(*t*), at different times. Although frequency information cannot be obtained from the phase space diagram, this method is useful for visualizing oscillatory processes, such as respiration. Amplitude of respiration was regarded as regular in visual analysis if the shape of the phase space diagram showed well defined, smooth margin ellipse and concordant patterns, just as in the case of phantom, as shown in Figure 2(b).

Second, respiration stability was assessed by analyzing the frequency distribution of the signal's power, the so-called power spectrum. The time-amplitude curves were transformed into the frequency domain by using the fast Fourier transform (FFT) algorithm [13, 14]. The most prominent spectral peak is called the fundamental frequency, representing the average frequency of patient's respiration. In visual analysis, the period of respiration was defined as regular if its corresponding spectrum satisfied the following conditions: (1) the spectrum contains one fundamental frequency peak, with its height exceeding the average of other peaks by at least twofold; (2) the spectrum is bell-shaped and centered around the fundamental frequency. In quantitative analysis, we measured the height of the fundamental peak (*A*_1_) to express the regularity of period.

A Poincaré map (or Poincaré section) captures the time series of a process in a phase space, where pairs of successive points in the time series define the points in the plot [14]. It has been often used to portray the dynamics of fluctuations between the intervals, such as in the studies of beat-to-beat heart rate variability [15–17]. We generated Poincaré sections by plotting the intersections of a given trajectory in the phase space diagram with a lower-dimensional state subspace, called the Poincaré plane, transverse to the trajectory [18, 19]. Herein, regarding the cross section location, we allowed a variation in both *v* and *x*, because if *x* or *v* is fixed, most of the respiratory signal will be lost because respiration of real patient has irregular and translational characteristics. So, we adopted the modified methods placing the Poincaré plane at the median point between the maximal and minimal points of respiration. A Poincaré map can be interpreted as a snapshot preserving the properties of the original trajectory. In this study, qualitative analysis of Poincaré plots was performed by visually inspecting the shapes formed by the points in the plots and evaluating the signals' regularity. Amplitude of respiration was regarded as regular if the shape of the Poincaré showed densely gathered pattern, just as in the case of phantom, as shown in Figure 2(c). However, a merely visual classification is insufficient because it is highly subjective in some equivocal cases. Hence, the plots were quantitatively analyzed by calculating the standard deviations (SD) along the horizontal (SD_*x*_) and vertical coordinates (SD_*v*_), for assessing the dispersion [14].

Finally, by calculating Lyapunov exponents, we evaluated dynamic and chaotic nature of respiration. Chaotic nature of respiration has been demonstrated previously by calculating the Lyapunov exponents for the data collected during the normal resting breathing of eight adults [20]. In this study, the largest Lyapunov exponents (LLEs) of 139 patients were calculated by using the previously suggested algorithm [21–25]. The LLE quantifies the expected divergence or convergence of initially close state-space trajectories as the system evolves in time [5]. The presence of a positive LLE is sufficient for diagnosing chaos and represents instability in a particular direction. The presence of a negative LLE represents the system's tendency to converge to a stable state. In the case of purely regular respiration, the LLE is 0. In this study, we compared the LLEs with temporal changes of the corresponding time-amplitude curves.

### 2.5. Statistical Analysis

One way analysis of variance (ANOVA) with Scheffe post hoc analysis and multivariant analysis of variance (MANOVA) were performed for the determination of statistically significant difference between the quantitative parameters among four groups, which were divided based on visual grading. In addition, receiver operating characteristic (ROC) curve analysis was conducted to define the quantitative cutoff value of amplitude (SD_*x*_, SD_*v*_), period (*A*_1_), and drift (MUD-MDD) for differentiating regular and irregular respiration. All* p* values were considered significant at <0.05. The Statistical Package for Social Sciences (version 16.0) and MedCalc (version 15.8) software were used for statistical analysis.

## 3. Results

**Figure 3:**
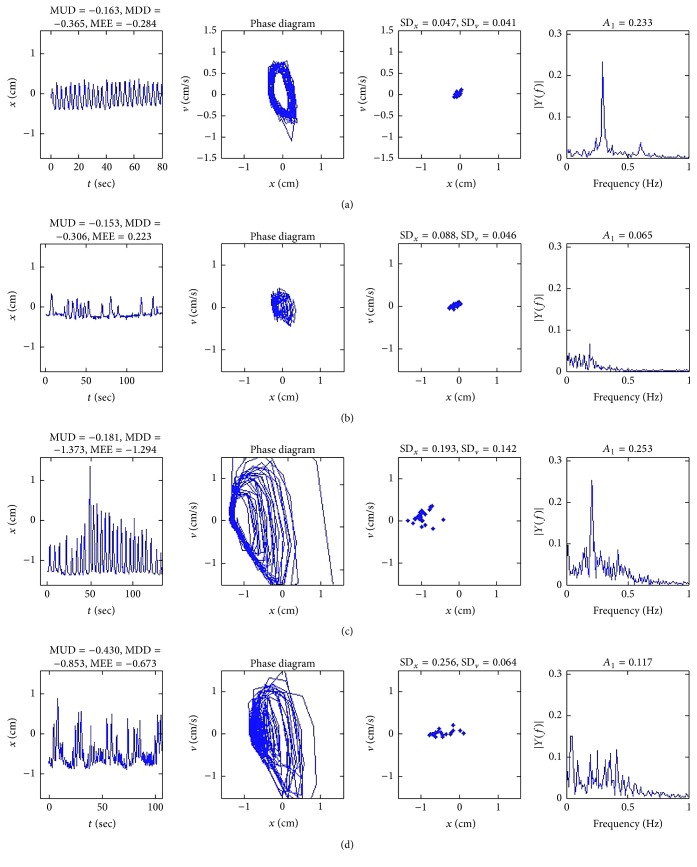
Representative respiratory patterns for Groups A (a), B (b), C (c), and D (d).

We classified the respiratory patterns of 139 patients into four groups according to the combination of regularity of amplitude and period, as shown in Table 2. As shown in Figure 3, respiratory patterns of 38 patients (Group A) exhibited regular amplitudes and periods. All patients in Group A did not show baseline drift (mean MUD-MDD was 0.242 ± 0.102). In respiratory patterns for Group A as shown in Figure 3(a), time-amplitude curves revealed regular amplitudes and frequencies and phase space diagrams showed shapes of constant form and size. Frequency spectra revealed narrow bell-shaped distributions around prominent fundamental frequency peaks (mean* A*_1_ was 0.178 ± 0.123). Poincaré maps revealed that the points formed densely gathered patterns (mean SD_*x*_ and SD_*v*_ were 0.068 ± 0.021, 0.053 ± 0.029).

In Group B, 23 patients exhibited regular amplitudes and irregular periods as shown in Figure 3(b). Only 4 patients showed baseline drift and 19 patients did not show baseline drift (mean MUD-MDD was 0.23 ± 0.88). Time-amplitude curves revealed regular amplitudes, but irregular frequencies. Phase space diagrams showed shapes of constant form and size. Poincaré maps revealed that the points formed densely gathered patterns (mean SD_*x*_ and SD_*v*_ were 0.071 ± 0.19, 0.035 ± 0.15). Frequency spectra revealed broader distributions with dispersive noise peaks (mean* A*_1_ was 0.078 ± 0.017).

In Group C, 54 patients exhibited irregular amplitudes, but regular periods as shown in Figure 3(c). 39 patients showed baseline drift and 15 patients did not show baseline drift (mean MUD-MDD was 0.445 ± 0.278). Time-amplitude curves revealed regular amplitudes, but irregular frequencies. Phase space diagrams showed irregular and discordant patterns. Poincaré maps revealed that the points were widely scattered (mean SD_*x*_ and SD_*v*_ were 0.151 ± 0.152, 0.091 ± 0.037). However, frequency spectra revealed broader distributions with dispersive noise peaks (mean* A*_1_ was 0.195 ± 0.097).

In Group D, 24 patients showed both irregular amplitude and periods as shown in Figure 3(d). All patients showed baseline drift (mean MUD-MDD was 0.793 ± 0.550). Time-amplitude curves revealed irregular amplitudes and frequencies. Phase space diagrams showed irregular and discordant patterns. Poincaré maps revealed that the points were scattered randomly without any direction (mean SD_*x*_ and SD_*v*_ were 0.251 ± 0.136, 0.095 ± 0.047). Frequency spectra revealed broader distributions with dispersive noise peaks (mean* A*_1_ was 0.121 ± 0.054).

There was statistically significant difference of quantitative parameters (SD_*x*_, SD_*v*_,* A*_1_, and MUD-MDD) among four groups (one way ANOVA, *p* = 0.0001 for MUD-MDD, SD_*x*_, and SD_*v*_, *p* = 0.0002 for* A*_1_) (Table 3). In Scheffe post hoc analysis, there were difference of MUD-MDD, SD_*x*_, and SD_*v*_ between A, B and C, D groups and difference of* A*_1_ between A, C and B, D groups. In addition, we conducted multivariant analysis of variance (MANOVA) to compare the difference of quantitative parameters (SD_*x*_, SD_*v*_,* A*_1_, and MUD-MDD) between groups depending on visual period, visual amplitude, and visual drift. Regarding visual period, there was difference of MUD-MDD (*F* = 5.242), SD_*x*_ (*F* = 3.954), and* A*_1_ (*F* = 26.109) between groups (Wilks' Lambda = 0.684, *p* = 0.0001). Regarding visual amplitude, there was difference of MUD-MDD (*F* = 11.005), SD_*x*_ (*F* = 16.326), and SD_*v*_ (*F* = 31.394) between groups (Wilks' Lambda = 0.741, *p* = 0.0001). However, the partial correlation revealed no significant visual period *∗* visual amplitude, visual period *∗* drift, visual amplitude *∗* drift, visual period *∗* visual amplitude *∗* drift interactions.

**Figure 4:**
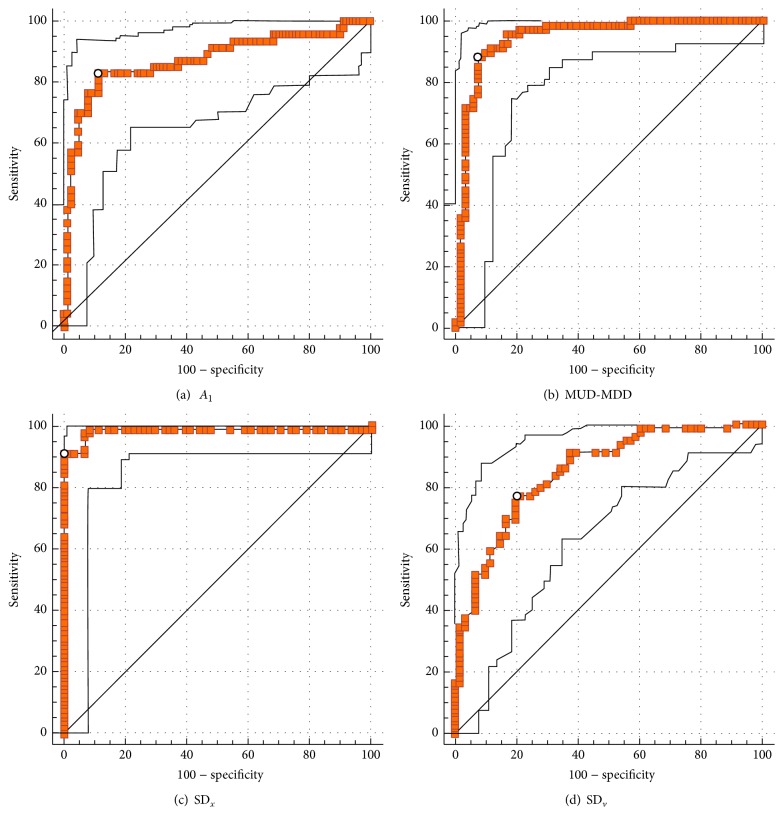
Receiver operating characteristic (ROC) curve analysis (red line) for quantitative parameters of* A*_1_ (a), MUD-MDD (b), SD_*x*_ (c), and SD_*v*_ (d). Black line on both sides of the ROC curve represents 95% confidence interval.

**Figure 5:**
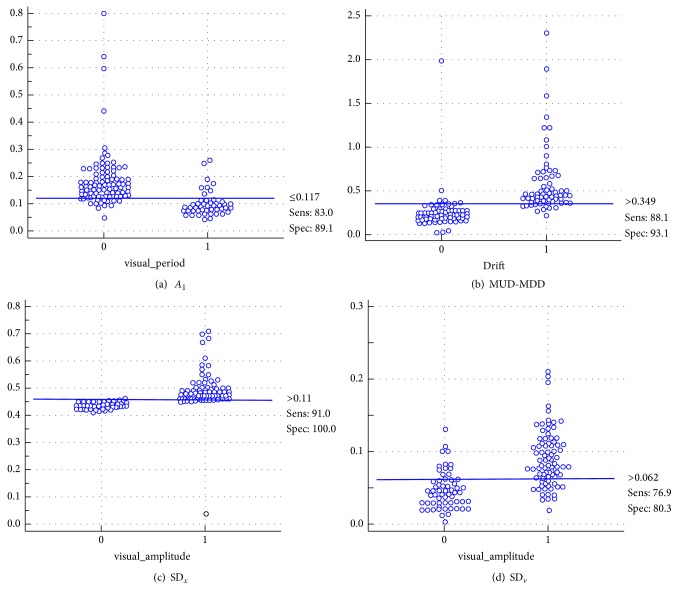
Interactive dot diagrams for quantitative parameters of* A*_1_ (a), MUD-MDD (b), SD_*x*_ (c), and SD_*v*_ (d).

Also, receiver operating characteristic (ROC) curve analysis was conducted to define the quantitative cutoff value of amplitude (SD_*x*_, SD_*v*_), period (*A*_1_), and drift (MUD-MDD) for differentiating regular and irregular respiration (Figures 4 and 5). Regarding binary visual analysis result as the reference of standard, the quantitative cutoff values for differentiating regular and irregular subcomponents of respirations were 0.11 for SD_*x*_ (AUC: 0.982, *p* < 0.0001), 0.062 for SD_*v*_ (AUC: 0.847, *p* < 0.0001), 0.117 for* A*_1_ (AUC: 0.876, *p* < 0.0001), and 0.349 for MUD-MDD (AUC: 0.948, *p* < 0.0001).

**Figure 6:**
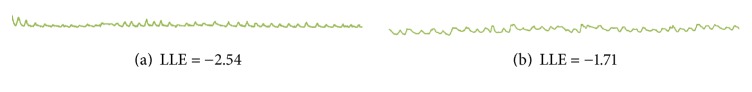
Temporal correlations of the time series with the corresponding LLEs.

Lastly, the data for all 139 patients exhibited negative LLEs, ranging from −3.76 to −0.43. (mean: −1.94, standard deviation: 0.46). The LLEs values were not correlated with the results of phase space, power spectrum, and Poincaré map analyses. A visual comparison of the time series with the LLEs (Figure 6) suggested that more negative Lyapunov exponents corresponded to faster regularization of initially irregular patterns.

## 4. Discussion

In this study, we have proposed both visual and quantitative methods for analyzing the stability of respiration during respiratory gated PET/CT. Using phase space diagrams, Fourier spectra, Poincaré maps, and Lyapunov exponents, we classified the respiratory patterns of 139 patients into four groups according to the combination of regularity of amplitude and period, as well as baseline position. Each group revealed characteristic shape and pattern on visual analysis, as well as showing statistically significant difference of the quantitative parameters between groups and quantitative cutoff value for differentiating regular and irregular respiration.

The advantages and limitations of the different methods are summarized in Table 4.

A specific advantage of the phase space diagram method is that it allows intuitively visualizing the oscillatory processes, such as respiration, at a glance. Phase space patterns are patient-specific. Compared with time-amplitude curve, this method becomes especially useful when the information on the respiration signal is acquired on a long time scale. However, quantitative analysis is difficult because a threshold for classifying regular and irregular respiratory patterns cannot be easily set by visually inspecting the phase space diagram. Moreover, in equivocal cases, which cannot be clearly categorized into regular or irregular, visual categorization is not easy. Finally, the analysis becomes difficult when the regularities of amplitude, frequency, and baseline are discordant or when there is a mixed pattern consisting of both regular and irregular respiratory patterns. In these cases, additional evaluation is required and other quantitative analysis methods, such as power spectrum or Poincaré map, can be helpful.

In the Fourier spectrum method, the information on amplitude and baseline position is lost during the Fourier transform. This method is useful for analyzing cases showing discordance between frequencies, amplitudes, or baseline positions (Group B2).

Complex cases that exhibit discordance between amplitudes and baseline positions (such as Groups B1, B2, and C) can be analyzed using the Poincaré map method. However, the main limitation of this method is that more than one Poincaré map can be generated, depending on the sectioning methods. In this study, Poincaré surfaces were positioned halfway between the maximal and minimal respiration points, because the median point was considered not to be affected by the fluctuations of extreme end-of-inhale or end-of-exhale points. In addition, the results of this analysis can be affected by the fitting method. In some studies, the shapes in Poincaré maps have been fitted to ellipses [26], while other studies have employed the Pearson correlation [27]. Finally, a number of techniques have been developed for quantifying the geometrical shapes in Poincaré maps. For example, a qualitative, visual classification method has been extended into a quantitative method by incorporating standard time-domain statistics into the existing categories of Poincaré plots [28, 29]. In our study, we adopted a simple way to express the extent of dispersion by measuring the standard deviation along the map horizontal coordinate.

Using the Lyapunov exponent method, we proposed a novel methodology for analyzing respiration waveforms by considering the respiratory system as a dynamical system. The LLEs for all 17 patients were negative, implying that, in all cases, the time series of breathing were attracted to the stable periodic orbits. By calculating the correlation between the time series and the corresponding Lyapunov exponents, we have shown that more negative exponents correspond to faster regularization of initially irregular respiratory patterns. In the future, the method of Lyapunov exponent will be especially useful in the clinical settings aiming to evaluate improvement of breathing pattern reproducibility using respiratory coaching.

The present study has some advantages over previous studies.

First, although other studies addressed different subcomponents of respiratory patterns, there was no attempt in the past to group the respiratory patterns into functionally distinct categories. Here, for the first time, we demonstrated that there were characteristic and distinctive shape and pattern on visual analysis and proved that there was significant difference of the quantitative parameters between groups.

Second, for the first time, we integrated both visual and quantitative approaches for evaluating the respiration stability. Our study proposed specific visual and quantitative criteria for classifying regular and irregular respiratory patterns. In the quantitative analysis methods (e.g., the power spectrum, the Poincaré map, and the Lyapunov exponent method), the extent of regularity was captured with continuous numerical variables. This is the first study providing quantitative indices of respiration stability and determining quantitative cutoff value for differentiating regular and irregular respiration.

Third, analysis in the previous studies was performed by decomposing the subcomponents of the respiration time-amplitude curve. However, this method cannot reveal a complete picture of the underlying complex breathing dynamics. In our study, the time-amplitude curves were transformed into various mathematical constructs, such as the phase space diagram, the Fourier spectrum, the Poincaré map, and the Lyapunov exponent. This study is the first to analyze multiple aspects of respiration using various methods of analysis, each of which has its advantages, limitations, and indications. Time-amplitude curves and phase space diagrams are sufficient for characterizing the respiration stability in the typical cases, such as those in Group A or Group D. However, when some inconsistency exists between the amplitudes, frequencies, and baseline positions (Group B or Group C), additional quantitative analysis tools, such as Fourier spectra and Poincaré maps, become helpful. The Fourier spectrum method is useful for classifying the respiration stability based on frequency. In contrast, Poincaré maps can be used for analyzing complex cases that exhibit baseline position drifts or a combination of regular and irregular respiratory patterns. The method of Lyapunov exponents can be used for revealing the temporal divergence or convergence of time series. Thus, these different methods are suitable for different clinical situations.

Several limitations of this study should be mentioned. First, visual grading system is binary system. Second, we used the respiration data acquired by using an external respiration-sensing monitor, which captures the anterior surface motion of the patient's abdomen. Although several studies have reported that the external marker represents internal motion, recent studies have shown a discrepancy between external respiratory motion and internal tumor motion [30]. Third, because this paper is focused on analyzing respiratory pattern only, potential research applications regarding the impact of the respiration patterns on PET/CT images have not been investigated.

The work presented in this paper can be extended in several ways. First, multiple visual grading systems can be applied for the visual analysis of phase space diagrams in the future studies. Second, other parameters, such as the difference between lesion SUVs or volumes of motion-free and motion-blurred PET/CT images, can be used as a reference for quantitatively determining the threshold. In the future work, we plan to investigate the impact of various respiratory patterns on the quantitative metabolic parameters, such as SUV and volume. Finally, it would be meaningful to investigate the changes in qualitative and quantitative indices of respiration stability that were mentioned in this study, before and after respiratory coaching in respiratory gated PET/CT.

These analysis methods can help design patient-specific respiratory methodologies. In respiratory gated PET/CT image, there is some change of SUV, compared with nongating image. Change of SUV between gating and nongated image can be due to either gating itself or effect of unstable respiration. In such situations, if we do not have information about patient's respiratory pattern, we cannot judge whether or not the changing SUV is due to gating itself or unstable respiration. So, to judge how we confide the changing SUV in the gated PET, we should evaluate the stability of respiration first. So, if we evaluate respiratory stability before acquiring PET/CT image, we can select patients showing unstable respiration and give those patients respiratory training before acquiring PET/CT.

## 5. Conclusions

In this study, we integrated both visual and quantitative methods for analyzing the stability of respiration during respiratory gated PET/CT using four methods: phase space diagrams, Fourier spectra, Poincaré maps, and Lyapunov exponents. Here, we demonstrated that each group revealed characteristic shape and pattern on visual analysis, as well as showing significant difference of the quantitative parameters between groups and determining quantitative cutoff value for differentiating regular and irregular respiration. These analysis methods can help design patient-specific respiratory methodologies in the future.

## Figures and Tables

**Table 1:** Baseline patient characteristics (*n* = 139).

Characteristics	Value
Gender	
Male	65
Female	74
Age	
Median	62
Range	44–81
Cancer type	
Stomach cancer	36
Pancreas cancer	13
Breast cancer	35
Lung cancer	30
Hepatocellular cancer	22
Carcinoma of unknown primary	3
Total	139

**Table 2:** Four categories of respiratory patterns of 139 patients based on visual analysis.

		Amplitude			
		Regular		Irregular	
Period	Regular	A (*n* = 38)		C (*n* = 54)	
		Drift yes	Drift no	Drift yes	Drift no
		0	38	39	15

Period	Irregular	B (*n* = 23)		D (*n* = 24)	
		Drift yes	Drift no	Drift yes	Drift no
		4	19	24	0

**Table 3:** Difference of quantitative parameters between groups: result of one way ANOVA and Scheffe post hoc analysis.

Quantitative parameters		*N*	Mean	SD	*p*	Scheffe
MUD-MDD	A	38	0.242	0.102	0.0001	A, B < C, D
	B	23	0.231	0.088		
	C	54	0.445	0.278		
	D	24	0.793	0.550		

SD_*x*_	A	38	0.069	0.021	0.0001	A, B < C, D
	B	23	0.071	0.019		
	C	54	0.151	0.152		
	D	24	0.251	0.136		

SD_*v*_	A	38	0.053	0.029	0.0001	A, B < C, D
	B	23	0.035	0.015		
	C	54	0.091	0.037		
	D	24	0.095	0.047		

*A*_1_	A	38	0.178	0.123	0.0002	A, C > B, D
	B	23	0.078	0.017		
	C	54	0.195	0.097		
	D	24	0.122	0.054		

**Table 4:** Advantages and limitations of phase space diagram, power spectrum, Poincaré map, and Lyapunov exponent methods.

	Advantage	Limitation
Phase space	(i) It allows intuitively visualizing the respiration stability.	(i) Quantitative analysis is difficult (threshold value cannot be set). (ii) Visual grading is difficult for equivocal patterns. (iii) Analysis becomes difficult when regularities of amplitude, frequency, and baseline position are discordant or when the respiratory pattern consists of regular and irregular patterns.

Power spectrum	(i) Stability is rated based on frequency. (ii) Both visual and quantitative analyses are possible.	(i) Information on amplitude and baseline position cannot be obtained.

Poincaré map	(i) It uses the shape and directionality of distribution patterns in Poincaré map as differential points and allows easy analysis of the cases in which the amplitude and baseline position are discordant. (ii) Both visual and quantitative analysis are possible.	(i) It cannot get information about frequency. (ii) Various Poincaré maps can be generated depending on various fitting and sectioning methods.

Lyapunov exponent	(i) Respiration is evaluated in terms of the temporal divergence or convergence.	(i) It is not correlated with phase space, power spectrum, and Poincaré map methods.
